# Diabetes and Suicide Risk Among Black Males, Including Sexual Minority Males: A Narrative Review With Primary Care Implications

**DOI:** 10.1007/s11892-026-01647-0

**Published:** 2026-09-28

**Authors:** Donte T. Boyd, Jaclynn Hawkins, Jamie Mitchell

**Affiliations:** 1 College of Social Work, The Ohio State University, 1947 N. College Road, 325K, Columbus, OH USA; 2 Department of Learning Health Sciences, Medical School, University of Michigan, Ann Arbor, MI USA; 3 School of Social Work, University of Michigan, Ann Arbor, MI USA

**Keywords:** Diabetes mellitus, Suicide, Black males, Black sexual minority males, Primary care, Health disparities

## Abstract

**Purpose of Review:**

Diabetes and suicide are intersecting public health concerns, yet evidence at this intersection remains sparse for Black males and especially Black sexual minority males (SMM). This focused narrative review synthesizes direct and adjacent evidence, with emphasis on diabetes-specific disparities, intersectional stress processes, and primary care implications.

**Recent Findings:**

The diabetes-suicide literature is heterogeneous, but consistently identifies depression, diabetes distress, treatment burden, complications, and access to insulin or other medications as clinically relevant pathways. For Black males, structural racism, economic inequity, health care discrimination, and constrained access to high-quality diabetes and mental health care may intensify these burdens. The most directly relevant study identified found more lifetime suicide attempts among African American adults with type 1 diabetes than controls, but did not report male-specific estimates. We identified no study estimating diabetes-related suicidal thoughts or behaviors specifically among Black SMM. Common tools such as the Patient Health Questionnaire-9, Diabetes Distress Scale, and Columbia-Suicide Severity Rating Scale have broad clinical support but have not been specifically validated in Black males with diabetes or Black SMM. Common tools such as the Patient Health Questionnaire-9, Diabetes Distress Scale, and Columbia-Suicide Severity Rating Scale have broad clinical support, but the literature identified in this review did not establish their population-specific validity among Black males with diabetes, including Black SMM.

**Summary:**

The central finding is an evidence gap, not proof of a multiplicative risk effect. Primary care clinicians should use culturally responsive psychosocial inquiry, direct suicide assessment when indicated, collaborative safety planning, and integrated behavioral health care while avoiding assumptions that screening tools perform equivalently across populations. Prospective, disaggregated research and population-specific measurement studies are urgently needed.

## Introduction

Diabetes affects approximately 40 million people in the United States, and Black communities continue to experience inequities in diabetes onset, treatment, complications, and mortality. [1, 2] These inequities are not properties of race itself. They reflect unequal exposure to social, economic, environmental, and health care conditions that shape the development and management of diabetes. At the same time, suicide rates among Black males have increased in recent decades, and discrimination, depression, hopelessness, and barriers to mental health care remain central prevention concerns [3–7]. 

Living with diabetes can add a distinct layer of psychological burden through daily self-management demands, diabetes distress, fear of complications, pain, functional loss, and access to potentially lethal medications [8–18]. These pathways may be especially consequential when diabetes is managed within settings marked by time constraints, fragmented behavioral health services, or racial discrimination. However, research rarely examines diabetes, suicidality, race, and male gender in the same study. Black sexual minority males are a subgroup of Black males who may encounter additional stressors related to sexual orientation, including heterosexism, identity concealment, family rejection, and stigma in health care [19–26]. Throughout this review, Black SMM refers to Black males who identify as gay, bisexual, queer, or another nonheterosexual identity, or who are classified in study-specific sexual minority samples on the basis of same-gender attraction or behavior. Sexual minority status is not treated as interchangeable with gender minority status.

Primary care is a critical setting for addressing these converging concerns because diabetes is often managed longitudinally in primary care and many people who die by suicide have recent contact with health services [27]. This review therefore asks a focused question: What is known about diabetes-related suicide risk among Black males and Black SMM, and what can primary care clinicians responsibly do given the limits of the evidence? We distinguish evidence that directly addresses this intersection from adjacent evidence that informs plausible pathways but cannot establish subgroup-specific prevalence, causality, or screening accuracy.

## Methods

We conducted a structured narrative review of peer-reviewed literature and authoritative clinical and public health sources published or updated from January 2000 through August 2026. PubMed/MEDLINE, PsycINFO, Web of Science, CINAHL, and the Cochrane Library were searched using combinations of terms related to diabetes, suicidal ideation, suicide attempt, suicide mortality, self-harm, Black or African American males, sexual minority males, primary care, screening, and intervention. Reference lists of relevant reviews and key empirical studies were also examined.

Because studies directly examining Black male identity, diabetes, and suicidality were expected to be limited, the synthesis was organized across five evidence streams: (1) direct studies of diabetes and suicide-related outcomes among Black participants; (2) general diabetes-suicide studies that clarify clinical mechanisms; (3) diabetes disparities and suicide research focused on Black males; (4) minority stress, cardiometabolic, and health care studies involving Black SMM; and (5) primary care screening and intervention research. We prioritized U.S. studies and findings disaggregated for Black males or Black SMM. Studies that combined racial, sex, or sexual-orientation groups without relevant subgroup estimates were used only as adjacent evidence and are identified as such. Given marked heterogeneity and the scarcity of direct studies, findings were synthesized narratively rather than pooled.

## Results

### Diabetes-Related Suicide Risk: Brief Context

Research linking diabetes with suicidality is heterogeneous rather than uniform. Reviews of type 1 diabetes (T1D) have reported elevated suicide risk in some cohorts but no consensus regarding its magnitude [8, 9]. A 2024 meta-analysis of adolescents and young adults with T1D found that suicidal ideation and attempts were clinically important, yet available comparisons did not clearly demonstrate higher prevalence than among peers without T1D because estimates were imprecise and highly heterogeneous [10]. For type 2 diabetes (T2D), depression and other psychiatric conditions are consistently associated with poorer diabetes outcomes and greater suicide risk, but findings vary by population, outcome definition, and duration of follow-up [6, 12, 13].

Across diabetes types, the most clinically relevant pathways include depression, diabetes-specific emotional distress, treatment fatigue, painful or disabling complications, fear of future morbidity, and disruption of social or occupational roles [11–17]. Diabetes distress is related to, but distinct from, depressive disorder and captures feeling overwhelmed, discouraged, unsupported, or burdened by the work of diabetes management [12–14]. For people who use insulin, access to a potentially lethal medication requires thoughtful assessment; however, insulin omission, inconsistent adherence, and glycemic instability should not be presumed to represent self-harm without direct clinical inquiry [15, 16].

This general literature provides context, but it does not answer the review question on its own. It supports diabetes-informed suicide assessment while also cautioning against blanket statements that every person with diabetes has the same level or type of risk. The sections that follow therefore center evidence specific to Black males and explicitly identify where conclusions rely on adjacent rather than direct studies.

### Diabetes-Specific Disparities and Suicide-Relevant Pathways Among Black Males

Black males experience a disproportionate burden of T2D and several diabetes-related complications, including kidney disease and lower-extremity morbidity. [1, 2, 28] These patterns should not be attributed to an inherent racial susceptibility. Contemporary evidence instead points to structural racism and its downstream consequences, including residential segregation, unequal educational and employment opportunity, environmental exposures, underinsurance, and differential access to preventive and specialty care [29–34]. A scoping review found that structural racism was associated with poorer glycemic and blood pressure outcomes, worse self-care indicators, lower standards of care, and higher mortality among adults with diabetes [30]. 

The daily work of diabetes management is also shaped by material conditions. Medication and food costs, transportation barriers, inflexible work schedules, unstable housing, limited access to safe spaces for physical activity, and competing family responsibilities can make recommended self-management routines difficult to sustain. [29, 32, 33] Socioeconomic and neighborhood conditions explain a substantial portion of racial inequities in T2D, although they do not account for all observed differences. [32, 33] When clinicians interpret these barriers as individual nonadherence, they may overlook how cumulative treatment burden can contribute to shame, frustration, diabetes distress, and hopelessness. The final link to suicide has not been established in Black male samples, but these are clinically plausible, modifiable pathways that warrant direct assessment rather than assumption.

Health care discrimination is another diabetes-specific pathway with particular relevance for Black males. A systematic review found that racism and discrimination in diabetes care were associated with communication problems, biased assumptions, poorer information sharing, and self-management behaviors that can worsen T2D outcomes among Black Americans [31]. In a study of 248 Black adults with T2D (163 females and 85 males), males reported more racial discrimination in health care than Black females, and perceived discrimination was associated with higher hemoglobin A1c among males but not females [34]. Black males also report inequities in patient-centered communication and opportunities for shared decision-making [35, 36]. These experiences can produce warranted mistrust, reduce continuity of care, and make patients less willing to disclose depression or suicidal thoughts 37– [36].

Racism can affect diabetes through both structural and stress-related processes. Chronic exposure to discrimination may activate neuroendocrine and inflammatory pathways, contribute to insulin resistance and glycemic dysregulation, and shape sleep, coping, health behaviors, and engagement with care. These processes should be understood as embodied consequences of chronic social stress rather than inherent racial biology [30, 31, 37]. Among older African American adults with T2D, perceived discrimination and lower physician trust partly explained higher diabetes distress [37]. Separate studies of Black males link racial discrimination with depression and suicidal or death ideation [5–7]. Taken together, these literatures support a pathway in which race-related stress may compound diabetes distress, but they do not establish that discrimination causes suicide among Black males with diabetes. The distinction between plausible mechanism and demonstrated causal pathway is essential.

Gendered expectations may further shape how distress is expressed and whether help is sought. Norms emphasizing self-reliance, stoicism, and control can make it difficult for some Black males to discuss emotional pain or ask for assistance with diabetes [38, 39]. These norms should not be treated as deficiencies in Black culture. Self-reliance, faith, family commitment, racial pride, and community responsibility can also function as strengths. Emerging diabetes research with Black males demonstrates the feasibility of peer-led, culturally tailored, and community-engaged approaches that prioritize trust, flexible delivery, and shared experience [40–42]. These models offer a stronger foundation for prevention than deficit-focused messaging.

The suicide context can be established briefly. Although suicide mortality remains lower among Black males than White males overall, the age-adjusted rate among non-Hispanic Black males rose to 13.1 per 100,000 in 2020, and more recent national data show significant increases among Black populations across several age groups [3, 4]. Research with Black males identifies racial discrimination, childhood adversity, depression, economic strain, and constrained access to culturally responsive mental health care as important contributors [5–7]. These trends strengthen the rationale for attention to diabetes-related distress, but they do not by themselves demonstrate a diabetes-specific effect.

These structural, clinical, and psychosocial pathways apply to Black males broadly. Black SMM should be understood within this population, not appended as a separate group. Their experiences may include the same diabetes and suicide-related burdens described above, together with additional stressors tied to sexual orientation. Integrating this perspective throughout the review avoids treating race, male gender, and sexual orientation as independent or interchangeable risk categories.

### Direct Evidence at the Diabetes-Suicide Intersection Among Black Males

The most directly relevant empirical study identified compared 412 African American adults with T1D with 404 African American control participants [43]. Lifetime suicide attempts were more common among participants with T1D than controls (13.3% vs. 3.5%). Within the T1D group, attempters were more likely to be female and to report depression, childhood trauma, hostility, smoking, and alcohol or drug misuse. The study is important because it directly examined diabetes and suicidality in an African American sample; however, it did not report suicide-attempt prevalence or effect estimates specifically for Black males. It therefore cannot establish the magnitude of diabetes-related suicide risk among Black males.

Beyond this study, we did not identify a population-based or prospective investigation reporting suicidal ideation, attempts, or suicide mortality specifically among Black males with diabetes. The current evidence does not support claims that the relative effect of diabetes on suicide mortality is larger for Black males than for White males, nor does it support a subgroup-specific prevalence estimate. The strongest conclusion is that clinically relevant risk processes converge in this population while direct epidemiologic evidence remains inadequate.

### Black Sexual Minority Males as a High-Priority Subgroup Within Black Males

Black SMM may encounter racism, heterosexism, identity concealment pressures, family rejection, housing or economic instability, and social isolation in ways that are mutually reinforcing rather than simply additive [19, 22–26]. National data show that gay and bisexual adults generally report more suicidal thoughts and behaviors than heterosexual adults across many racial and ethnic groups, although subgroup estimates vary [19]. Within Black SMM samples, intersectional discrimination is associated with psychological distress, depression, and other syndemic conditions [22, 25]. Recent studies of Black gay and bisexual male adolescents and young Black SMM also link anticipated discrimination with suicidal behaviors and identify positive identity and other developmental assets as potential sources of protection [20, 21].

Health care stigma may intensify these concerns. In a mixed-methods study of Black SMM, anticipated health care stigma was associated with substantially lower use of HIV pre-exposure prophylaxis, and interviews described identity-specific stigma, socioeconomic assumptions, and sexual-risk stereotyping [24]. Although this study did not involve diabetes care, it demonstrates how prior or anticipated stigma can shape engagement with preventive services. In diabetes settings, the same dynamics may affect continuity, disclosure of distress, and willingness to discuss suicidal thoughts. This is an inference from adjacent evidence and should be tested directly.

Evidence on sexual orientation and diabetes is mixed and rarely disaggregated by race. In a nationally representative study, bisexual behavior was associated with prevalent diabetes, whereas sexual minority identity and attraction were not [26]. This finding underscores that identity, attraction, and behavior are distinct dimensions and should not be collapsed. Available studies do not justify the claim that all Black SMM have elevated diabetes prevalence, and they provide no basis for estimating diabetes-related suicide risk within this subgroup.

We identified no study that directly estimated suicidal ideation, suicide attempts, or suicide mortality among Black SMM with diabetes. Black SMM are therefore best framed as a high-priority subgroup because multiple established risk processes may converge, not because a multiplicative or “triple jeopardy” effect has been empirically demonstrated. This distinction strengthens the review by pairing urgency with appropriate evidentiary restraint.

### Primary Care Implications: Identification, Assessment, and Response

Primary care offers repeated opportunities to identify diabetes distress, depression, and suicide risk, yet short visits, competing clinical demands, limited behavioral health training, and weak referral pathways can impede meaningful assessment [27–46]. These barriers may be amplified for Black males and Black SMM when clinicians fail to recognize racism-related stress, medical mistrust, or sexual-orientation-related stigma. The 2026 American Diabetes Association Standards of Care emphasize person-centered psychosocial care, culturally and socially responsive diabetes self-management support, and attention to social determinants [46]. Suicide prevention can be incorporated into this broader model rather than treated as a separate task.

The Patient Health Questionnaire-9 (PHQ-9), Diabetes Distress Scale (DDS), and Columbia-Suicide Severity Rating Scale (C-SSRS) are reasonable evidence-supported tools, but their population-specific performance among Black males with diabetes, including Black SMM, was not established by the literature identified in this review. The PHQ-9 has broad validation, and a large multisystem study found statistically detectable but clinically negligible differential item functioning across broad racial and ethnic categories [47, 48]. That study did not establish performance specifically for this population. The DDS demonstrated reliability and validity across diverse clinical sites, but the literature identified in this review did not establish measurement invariance or cut points for this population [13, 14]. The C-SSRS has psychometric and predictive support in mixed clinical samples, yet we identified no validation study focused on Black males with diabetes, including Black SMM [49, 50].

Accordingly, these instruments should structure, not replace, a culturally responsive clinical conversation. A positive response to PHQ-9 item 9 requires direct follow-up about suicidal thoughts, intent, plans, past behavior, and current safety; a negative item 9 response should not end assessment when other warning signs are present. Clinicians should ask behaviorally specific questions, explore diabetes distress separately from depression, and inquire about racism, mistrust, sexual minority stress, social support, and reasons for living. For patients using insulin or other potentially lethal medications, means-safety planning should be collaborative and individualized so that suicide prevention does not become punitive restriction that compromises diabetes care.

**Fig. 1:**
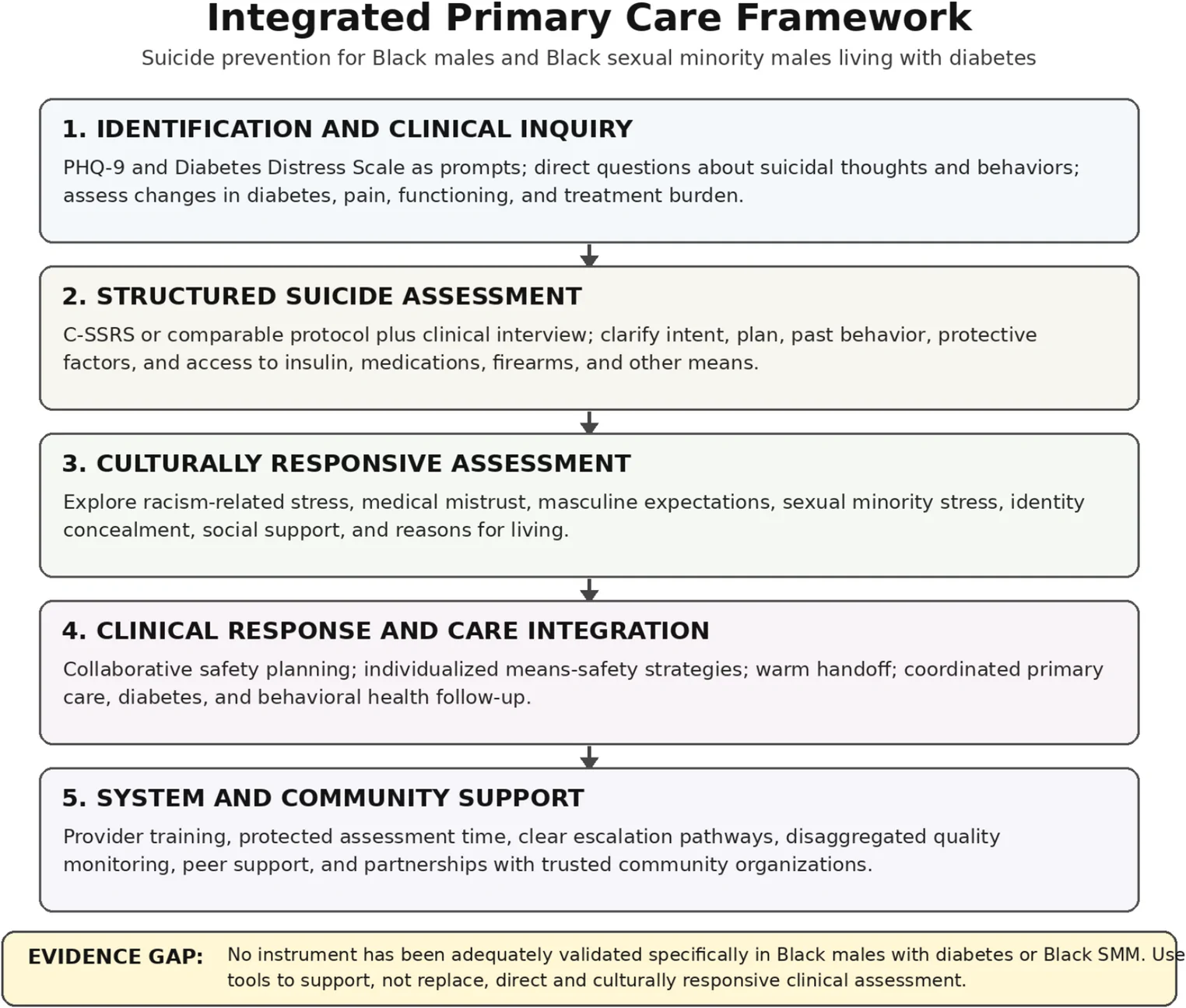
Integrated primary care framework for suicide prevention among Black males and Black sexual minority males with diabetes. General instruments such as the PHQ-9, DDS, and C-SSRS can support identification and assessment, but the literature identified in this review did not establish their population-specific validity among Black males with diabetes, including Black SMM. The framework therefore pairs structured tools with direct clinical inquiry, culturally responsive and sexual-orientation-affirming assessment, collaborative safety planning, integrated behavioral health care, and community or system supports. Solid arrows indicate the clinical pathway; the evidence-gap bar applies across all levels

Figure 1 presents an integrated primary care framework built around five linked functions: initial identification, structured suicide assessment, culturally responsive inquiry, evidence-based clinical response, and system or community support. The framework makes the measurement limitation explicit and positions clinical judgment, relationship quality, and warm handoffs as essential rather than optional.

## Discussion

The most important finding of this review is the scarcity of direct evidence. Diabetes-related suicide risk has been studied in general populations, diabetes disparities have been studied among Black adults, and suicidality has been studied among Black males and Black SMM. These literatures identify meaningful points of convergence, but they cannot be combined to produce a subgroup-specific risk estimate. The review therefore supports heightened clinical attention without claiming that diabetes creates a proven multiplicative effect on suicide risk among Black males or Black SMM.

For Black males, the central argument is structural and diabetes-specific. Unequal access to high-quality care, economic and neighborhood constraints, health care discrimination, and the cumulative stress of racism can increase treatment burden, worsen glycemic outcomes, and intensify diabetes distress [29–36]. These pathways are more informative than a generic description of diabetes and suicide in the overall population. They also point to modifiable targets: respectful communication, shared decision-making, affordable treatment, attention to social needs, and continuity with clinicians who earn trust.

Black SMM belong within this analysis of Black males while also requiring explicit attention to sexual minority stress. The available evidence supports concern about intersectional discrimination, health care stigma, depression, and suicidality, but does not establish that Black SMM have uniformly higher diabetes prevalence or a quantified diabetes-suicide effect [19–26]. Framing this subgroup consistently throughout the review makes the argument more coherent and avoids conflating sexual orientation with gender identity.

Primary care recommendations must be equally precise The PHQ-9, DDS, and C-SSRS have value, but the literature identified in this review did not establish their population-specific validity among Black males with diabetes, including Black SMM. Structured instruments are most useful when paired with direct, empathic questions and an understanding of how diabetes, racism, masculinity, sexual minority stress, and prior health care experiences shape disclosure. This approach reduces the risk that screening becomes a checklist detached from the patient’s lived context.

Integrated care is especially well suited to these concerns. Safety Planning Intervention, warm handoffs, collaborative care, and community-engaged diabetes support can connect acute suicide prevention with ongoing diabetes management [40–42, 51, 52]. Implementation should preserve access to medically necessary insulin and other treatments while addressing lethal-means safety collaboratively. Trusted peer, faith, family, and community supports may strengthen engagement when they are selected by the patient rather than imposed by the health system.

## Limitations and Future Research Directions

Several limitations shape interpretation. First, this is a focused narrative review rather than a systematic review with a pooled effect estimate; although the search was structured and updated through August 2026, study selection and synthesis were necessarily guided by relevance to a highly specific question. Second, direct evidence was extremely limited. The most relevant African American T1D study did not report male-stratified estimates, and no identified study directly examined diabetes-related suicidality among Black SMM [43]. Much of the synthesis therefore relies on adjacent, often cross-sectional literatures. These studies can identify plausible mechanisms but cannot establish causal or multiplicative effects. Third, diabetes type, treatment regimen, age, suicidal outcome, and follow-up period varied widely, limiting comparisons.

Measurement limitations are also substantial. Race, sex, gender, sexual identity, attraction, and behavior are inconsistently collected and frequently collapsed, which can erase meaningful within-group differences. The literature identified in this review did not establish the population-specific validity of the PHQ-9, DDS, and C-SSRS among Black males with diabetes, including Black SMM. This evidence gap does not establish whether sensitivity, specificity, or cut-point performance differs from that observed in other populations. Future studies should use prospective designs, link clinical and mortality data, oversample Black males and Black SMM, disaggregate sexual-orientation dimensions, distinguish T1D from T2D, and test measurement invariance and predictive validity. Intervention research should be co-designed with patients and community partners and should evaluate both metabolic and suicide-related outcomes.

## Clinical Recommendations

Primary care and diabetes teams should incorporate periodic assessment of emotional well-being and diabetes distress, with direct suicide inquiry when symptoms, clinical changes, functional losses, medication concerns, or patient statements raise concern [46]. The PHQ-9 and DDS can help open these conversations, and the C-SSRS or a comparable structured protocol can support a fuller assessment. Clinicians should recognize the limitations of population-specific validation evidence and interpret scores alongside direct clinical inquiry and the patient’s context, rather than relying on a single score or cut point.

Assessment should be culturally responsive and sexual-orientation affirming. This includes asking how racism, discrimination, mistrust, masculine expectations, identity concealment, family responses, financial strain, and prior health care experiences affect diabetes management and emotional well-being. Clinicians should not assume heterosexuality, should use the patient’s own language for identity and relationships, and should recognize cultural identity, spirituality, family, peers, and community connection as potential sources of protection.

When suicide risk is identified, clinicians should use a collaborative Safety Planning Intervention, make a warm handoff to behavioral health care when available, and arrange timely follow-up [51, 52]. Means-safety discussions should include insulin, oral medications, firearms, and other accessible methods, but changes to diabetes treatment should be made jointly with the patient and diabetes team. Abrupt or punitive restriction of insulin can undermine trust and create medical danger.

Health systems should support these practices through integrated behavioral health services, protected time for assessment, clear escalation pathways, provider training, and routine review of disparities in screening, referral, and follow-up. Data systems should support disaggregation within Black male populations by voluntarily reported sexual orientation, while protecting privacy. Partnerships with Black community organizations and organizations serving Black SMM can strengthen trust, referral options, and culturally grounded support. Measurement research should be treated as an implementation priority rather than deferred indefinitely (Table 1).

**Table 1:** Integrated strategies for suicide prevention among Black males with diabetes, including Black sexual minority males

Level of care	Focus	Illustrative strategies
Identification	Detect distress and possible suicide risk	Use the PHQ-9 and DDS as conversation prompts; ask direct, behaviorally specific suicide questions when indicated; do not rely on a single score or cut point.
Structured assessment	Clarify severity, immediacy, and context	Use the C-SSRS or a comparable protocol alongside clinical interview; assess intent, plan, past behavior, protective factors, and access to insulin, medications, firearms, and other means.
Clinical response	Address acute and ongoing risk	Use collaborative Safety Planning Intervention, individualized means-safety planning, timely follow-up, and emergency escalation when clinically necessary.
Cultural responsiveness	Improve trust, disclosure, and fit	Assess racism-related stress, medical mistrust, masculine expectations, and sexual minority stress; use culturally responsive and sexual-orientation-affirming communication.
Care integration	Coordinate metabolic and behavioral health care	Use warm handoffs, co-located or collaborative care, shared treatment planning, and communication between primary care, diabetes, and behavioral health teams.
Community and systems	Sustain prevention beyond the visit	Partner with Black community organizations and organizations serving Black SMM; support peer-led care, provider training, protected assessment time, and equity-focused quality improvement.
Measurement equity	Address the evidence gap	Validate the PHQ-9, DDS, and C-SSRS among Black males with diabetes, including Black SMM; evaluate measurement invariance, cut points, false-positive and false-negative patterns, and predictive validity.

Note. PHQ-9 = Patient Health Questionnaire-9; DDS = Diabetes Distress Scale; C-SSRS = Columbia-Suicide Severity Rating Scale. These tools are evidence-supported in broader populations, but the literature identified in this review did not establish their population-specific validity among Black males with diabetes, including Black SMM. They should be used with direct clinical inquiry and culturally responsive judgment rather than as stand-alone determinants of risk

## Conclusion

Diabetes and suicide-related risk processes converge in clinically important ways for Black males, yet direct subgroup-specific evidence remains strikingly limited. Structural racism, material hardship, discrimination in health care, diabetes distress, depression, complications, and barriers to culturally responsive mental health services create a credible basis for concern. Black SMM should be understood as a subgroup within Black males who may also experience sexual minority stress and health care stigma, not as an interchangeable label for all Black or LGBTQ+ people.

Primary care can respond now without overstating the science. Clinicians can ask directly about emotional distress and suicide, use structured tools cautiously, address insulin and medication access collaboratively in safety planning, and connect patients to integrated behavioral health and trusted community supports. These actions are strongest when grounded in cultural humility, sexual-orientation-affirming care, and attention to the structural conditions that make diabetes management more difficult.

The research agenda is equally clear: generate prospective, disaggregated evidence; validate screening and assessment tools in Black males with diabetes and Black SMM; and co-design interventions that measure both metabolic and suicide-related outcomes. Until that evidence exists, the responsible position is neither inaction nor overstatement, but careful, equity-focused clinical vigilance.

## Data Availability

The data and materials used in this study are available from the author upon reasonable request.
